# Impact of the Preoperative C-reactive Protein to Albumin Ratio on the Long-Term Outcomes of Hepatic Resection for Intrahepatic Cholangiocarcinoma

**DOI:** 10.31557/APJCP.2020.21.8.2373

**Published:** 2020-08

**Authors:** Tokuji Ito, Hiroji Shinkawa, Shigekazu Takemura, Shogo Tanaka, Takayoshi Nishioka, Toru Miyazaki, Atsushi Ishihara, Shoji Kubo

**Affiliations:** *Department of Hepato-Biliary-Pancreatic Surgery, Osaka City University Graduate School of Medicine, 1-4-3 Asahimachi, Abenoku, Osaka, 545-8585, Japan. *

**Keywords:** CRP/Alb ratio, intrahepatic cholangiocarcinoma, hepatic resection, prognostic factor

## Abstract

**Objective:**

The present study aimed to investigate the impact of preoperative C-reactive protein to albumin (CRP/Alb) ratio on the long-term outcomes of patients with intrahepatic cholangiocarcinoma (ICC).

**Methods:**

82 patients who underwent hepatic resection for mass-forming type of ICC were evaluated. The relationship between preoperative CRP/Alb ratio and survival outcomes was investigated.

**Results:**

The optimal cutoff value of CRP/Alb ratio for assessing overall survival (OS) was determined as 0.089. Univariate analysis for recurrence-free survival (RFS) showed that CRP/Alb ratio >0.089, carbohydrate antigen 19-9 (CA 19-9) >37 U/mL, lymph node metastasis, vascular invasion, and multiple tumors were significantly associated with postoperative recurrence. On multivariate analysis, the independent prognostic factors identified were CRP/Alb ratio >0.089 (p < 0.001), lymph node metastasis (p = 0.006), and multiple tumors (p < 0.001). Univariate analysis for OS showed that CRP/Alb ratio >0.089, CA 19-9 >37 U/mL, lymph node metastasis, vascular invasion, multiple tumors, and positive surgical margin were significantly associated with overall death. On multivariate analysis, the independent prognostic factors identified were CRP/Alb ratio >0.089 (p < 0.001), lymph node metastasis (p = 0.01), and multiple tumors (p = 0.005).

**Conclusion:**

Preoperative CRP/Alb ratio may predict poor long-term outcomes after hepatic resection in patients with ICC.

## Introduction

Intrahepatic cholangiocarcinoma (ICC), which arises from the bile ducts in the liver, is the second most common form of primary hepatic cancer after hepatocellular carcinoma (Shirabe et al., 2010; Farges et al., 2011; Zou et al., 2014; Siegel et al., 2015; Bridgewater et al., 2014). The incidence of ICC in East Asia is the highest in the world; however, recent nationwide population-based studies have shown an increasing incidence of ICC in western countries (Petrick et al., 2016). Currently, surgery is the only potentially curative treatment option for ICC. Despite the recent advances in surgical techniques, chemotherapy, and radiotherapy, the survival outcome after surgery remains poor, with a five-year survival rate of approximately 30% (Farges et al., 2010). Therefore, useful markers for stratifying the prognosis of patients with ICC are needed to develop appropriate treatment strategies that can improve survival outcomes after surgery.

Recently, there has been increasing evidence that systemic inflammatory response predicts the survival outcome in cancer patients. C-reactive protein (CRP), which is one of the inflammatory markers, is an acute-phase reactant that is synthesized by hepatocytes (Morris-Stiff et al., 2008; Nakazaki, 1992). Previous studies have demonstrated that an increased level of CRP is associated with worse prognosis in patients with various kinds of tumors (Nozoe et al., 1998; Hashimoto et al., 2005; Pathak et al., 2014; Ishino et al., 2014; Shrotriya et al., 2015). Further, serum albumin (Alb), which is the traditional standard marker of nutritional status, is also known to reflect inflammatory status (Ishida et al., 2014). Combination of these inflammatory markers into a CRP/Alb ratio had been demonstrated as a prognostic indicator in several kinds of cancers, such as colorectal (Ishizuka et al., 2016; Shibutani et al., 2016), gastric (Saito et al., 2018), esophageal (Wei et al., 2015), hepatocellular (Kinoshita et al., 2015), and pancreatic (Haruki et al., 2016; Liu et al., 2017). Therefore, we believe that the CRP/Alb ratio may predict the postoperative prognosis of patients with ICC.

In this study, we retrospectively investigated the relationship between preoperative CRP/Alb ratio and survival outcomes in patients who underwent hepatic resection for ICC.

## Materials and Methods

*Patients*

The medical records of 82 patients who underwent initial and curative hepatic resection for a mass-forming (MF) type of ICC at the Osaka City University Hospital between January 1998 and October 2017 were retrospectively reviewed. In this study, ICC was pathologically defined as cholangiocarcinoma centering in second or more peripheral branches of the intrahepatic bile ducts. There were no patients with jaundince, cholangitis or bile duct drainage. ICC tumors were classified according to the macroscopic classification proposed by the Liver Cancer Study Group of Japan (Liver Cancer Study Group of Japan, 2010). Patients with pure intraductal growth or pure periductal-infiltrating type of ICC were excluded because these types of ICC were known to have different biological behaviors from that of MF-dominant ICC (Shimada et al., 2007; Yamamoto et al., 2009; Uno et al., 2012). Curative resection was defined as the complete removal of the entire macroscopic tumor without residual tumors. All analyses were performed in accordance with the ethical guidelines for clinical studies at Osaka City University Hospital (approval number, 3815). Comprehensive informed consent to use patient information for this study was obtained from the patients before surgery. 

*Clinical data extraction*

We collected the baseline characteristic of the participants, including age, gender, and disease stage, which was determined based on the Eighth Edition of the American Joint Committee on Cancer staging criteria. All blood test values recorded in this study were obtained within 2–4 days before the operation and included CRP, Alb, alanine aminotransferase (ALT), carbohydrate antigen 19-9 (CA 19-9), and carcinoembryonic antigen (CEA). The CRP/Alb ratio was calculated by dividing the serum CRP level by the serum Alb level.

*Patient follow-up*

After hepatic resection, all patients were regularly screened for recurrence by monitoring the plasma levels of tumor markers (CEA and CA 19-9), ultrasonography, and dynamic computed tomography. Recurrence was defined as the appearance of a new tumor lesion that had radiological features of ICC. When recurrence was detected, the patient received further treatment like repeated hepatic resection or others, such as chemotherapy. The period of follow-up was from the date of the initial surgery until March 30, 2018 or death from any cause. Patients who did not die at the time of last follow-up were censored.

*Statistical analysis*

All statistical analyses were performed using EZR software (Saitama Medical Center, Jichi Medical University, Saitama, Japan), which is the graphical user interface for R (The R Foundation for Statistical Computing, Vienna, Austria) (Kanda, 2013). Categorical variables were compared using the Fisher exact probability method or *χ*^2^ test. Continuous variables were presented as median (range). The Mann–Whitney U-test was performed to evaluate the differences between the two groups. The optimal cutoff level of the CRP/Alb ratio was determined by a web-based system that was engineered by the R software (http://molpath.charite.de/cutoff) (Budczies et al., 2012). Survival curves were calculated according to the Kaplan–Meier method. Univariate analyses were conducted using the log-rank test. Multivariate analyses of the variables that affected survival were performed using the Cox proportional hazards model. P-values were derived from two-tailed tests, and statistical significance was set at <0.05.

The variables that were potentially associated with postoperative prognosis were selected based on the results of a previous study and on our own clinical experience (Uenishi et al., 2014; Spolverato et al., 2015; Pan et al., 2017). These included age (≤65 or >65 years); sex; body mass index (≤25 or >25 kg/m2); hepatitis virus infection; CRP (≤0.4 or >0.4 mg/dL); Alb concentration (≤4.0 or >4.0 g/dL); ALT activity (≤30 or >30 IU/L); liver cirrhosis, CEA (≤5 or >5 ng/mL); CA 19-9 (≤37 or >37 U/mL); largest diameter of the main tumor (>5.0 or ≤5.0 cm); single or multiple tumors; presence of intrahepatic metastases, lymph node metastasis, bile duct tumor invasion, or microscopic vascular invasion; surgical margin; and adjuvant chemotherapy.

## Results

*Clinicopathological profiles of the patients*

The clinicopathological characteristics of the patients are detailed in Supplementary Table 1. The number of patients classified as stage I, II, III, and IVa were 21, 23, 15, and 23, respectively. Of all patients, 29 (35.4%) had hepatitis virus infection, and 9 (11%) had alcoholic hepatitis. The overall median follow-up duration was 43.1 months (range, 2–127.5 months). During the study period, 50 patients (61%) had postoperative recurrence, and 45 patients (54.9%) died. 

*Cutoff value of the CRP/Alb ratio*

**Figure 1:**
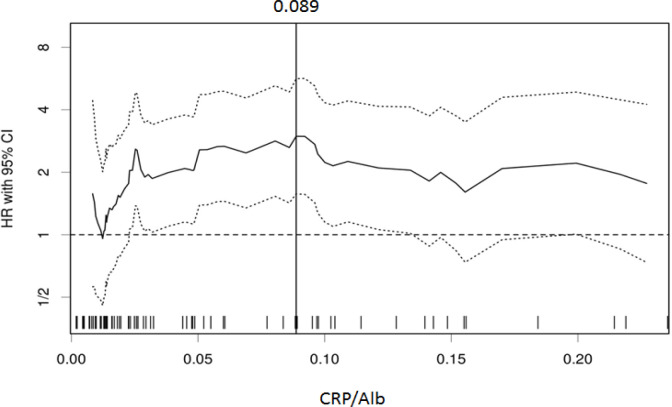
Association between Preoperative CRP/Alb Ratio and Overall Survival of Patients with Intrahepatic Cholangiocarcinoma. The hazard ratio (HR) including the 95% confidence interval (CI) is plotted in relation to the cutoff values. The vertical line designates the dichotomization showing the most significant correlation with overall survival

Using the biostatistical tool, Cutoff Finder, we found a wide range of cutoff values for the CRP/Alb ratio and determined 0.089 as the optimal value for assessing overall survival (OS) (Figure. 1). Patients were divided into two groups based on the cutoff value of the CRP/Alb, as follows: the CRP/Alb ratio >0.089 group (n = 26) and the CRP/Alb ratio ≤0.089 group (n = 56).

*Relationship between the preoperative CRP/Alb ratio and the clinicopathological features of patients with ICC*

Table 1 shows the clinicopathological characteristics of the patients based on the CRP/Alb ratio. Compared with the CRP/Alb ratio ≤0.089 group, the CRP/Alb ratio >0.089 group comprised significantly more patients with tumor size >5 cm (50% vs. 21.4%, p = 0.019) and bile duct tumor invasion (65.4% vs. 39.3%, p = 0.034). Microvascular invasion tended to be frequently observed in in the CRP/Alb >0.089 group than the ≤ 0.089 group (61.5% vs. 37.5%, p = 0.057).

*Recurrence-free survival (RFS) and OS*

**Figure 2:**
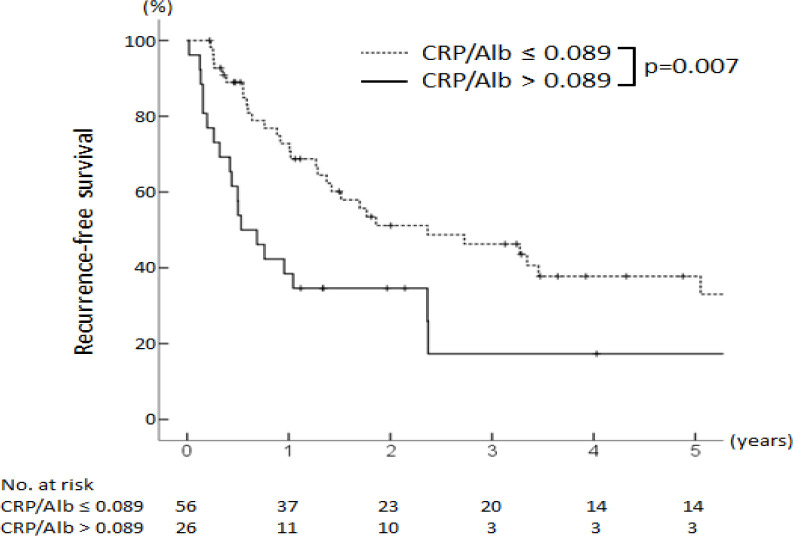
Kaplan-Meier Analysis of the Recurrence Free Survival of Intrahepatic Cholangiocarcinoma Patients with a High (>0.089) vs Those with a Low (≤0.089) CRP/Alb Ratio. There were significant difference between the groups

**Figure 3:**
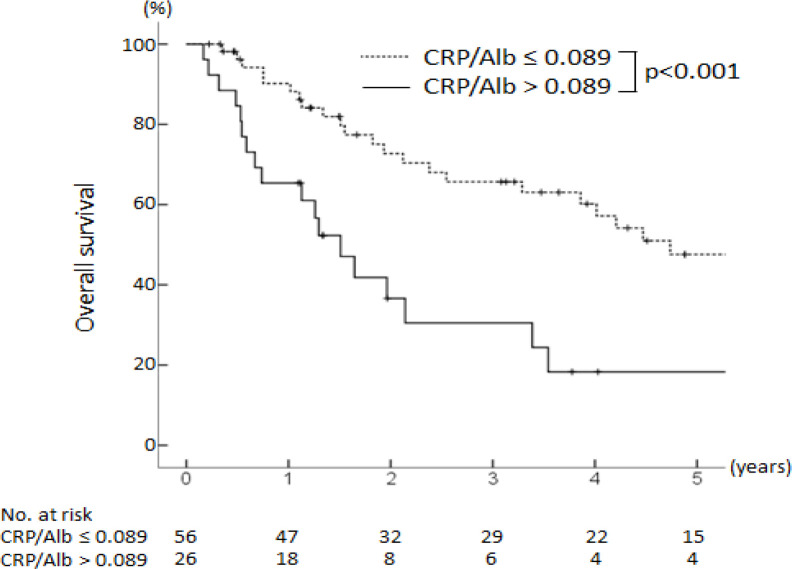
Kaplan-Meier Analysis of the Overall Survival of Intrahepatic Cholangiocarcinoma Patients with a High (>0.089) vs Those with a Low (≤0.089) CRP/Alb Ratio. There were significant difference between the groups

The respective one-, three-, and five-year RFS rates after surgery were 39%, 17%, and 17% in the CRP/Alb ratio >0.089 group and 73%, 46%, and 38% in the CRP/Alb ratio ≤0.089 group (p = 0.007) (Figure 2). The respective one-, three-, and five-year OS rates after surgery were 65%, 31%, and 18% in the CRP/Alb ratio >0.089 group and 90%, 66%, and 48% in the CRP/Alb ratio ≤0.089 group (p < 0.001) (Figure 3).

*Prognostic factors for RFS among patients with ICC after hepatic resection*

Table 2 shows the prognostic factors for RFS. According to the univariate analysis, the CRP/Alb ratio >0.089, CA19-9 >37, lymph node metastasis, vascular invasion, and multiple tumors were significantly associated with postoperative recurrence. The independent prognostic factors for RFS, according to the multivariate analysis, were CRP/Alb ratio >0.089 [hazard ratio (HR), 3.00; 95% confidence interval (CI), 1.58–5.69; p <0.001]; lymph node metastasis (HR, 2.64; 95% CI, 1.32–5.30; p = 0.006); and multiple tumors (HR, 3.34; 95% CI, 1.69–6.61; p <0.001). 

*Prognostic factors for OS among patients with ICC after hepatic resection*

Table 3 shows the prognostic factors for OS. According to the univariate analysis, the CRP/Alb >0.089, CA19-9 >37, lymph node metastasis, vascular invasion, multiple tumors, and surgical margin positive were significantly associated with OS. The independent prognostic factors for OS, according to the multivariate analysis, were CRP/Alb ratio >0.089 (HR, 3.39; 95% CI, 1.72–6.67; p < 0.001); lymph node metastasis (HR, 2.82; 95%, CI 1.29–6.19; p = 0.01); and multiple tumors (HR, 2.70; 95% CI, 1.34–5.45; p = 0.005).

**Table 1:** Comparison of Patients with Intrahepatic Cholangiocarcinoma Categorized by C-Reactive Protein to Albumin Ratio

Variable	CRP/Alb >0.089 (n=26)	CRP/Alb ≤0.089 (n=56)	*p*-value
Gender (Male/Female)	18/8	42/14	0.6
Age (years)	67.5 (49-82)	68 (32-82)	0.78
Age >65 (years)	16 (61.5%)	32 (57.1%)	0.81
BMI >25	8 (30.8%)	17 (30.4%)	>0.99
Hepatitis Virus presence	6 (23.1%)	23 (41.2%)	0.14
ALT >30 (IU/L)	10 (38.5%)	24 (42.9%)	0.81
CEA >5 (ng/mL)	9 (52.9%)	17 (30.3%)	0.8
CA19-9 >37 (U/mL)	14 (53.8%)	24 (42.9%)	0.48
Liver cirrhosis	2 (8.3%)	9 (16.1%)	0.49
TNM classification (III-IVa)	13 (50%)	25 (44.6%)	0.81
Lymph node metastasis	5 (23.8%)	13 (23.2%)	0.78
Bile duct invasion	17 (65.4%)	22 (39.3%)	0.034
Microvascular invasion	16 (61.5%)	21 (37.5%)	0.057
Multiple tumor	6 (23.1%)	13 (23.2%)	>0.99
Tumor size >5 (cm)	13 (50%)	12 (21.4%)	0.019
Surgical margin positive	5 (23.8%)	7 (12.5%)	0.51
Adjuvant chemotherapy	10 (38.5%)	31 (55.4%)	0.24

CRP/Alb, C-reactive protein/albumin; BMI, body mass index; ALT, alanine aminotransferase; CEA, carcinoembryonic antigen; CA19-9, carbohydrate antigen 19-9; TNM, tumor-node-metastasis

**Table 2:** Prognostic Factors for Recurrence-Free Survival

Variable		Univariate		Multivariate	
	N	HR (95% CI)	p-value	HR (95% CI)	p-value
Gender					
Male	60	0.59(0.32-1.07)	0.083		
Female	22				
Age (year)					
>65	48	1.37(0.77-2.44)	0.29		
≤65	34				
BMI (kg/m^2^)					
>25	25	1.05(0.58-1.89)	0.88		
≤25	57				
Hepatitis virus infection					
Presence	29	1.17(0.65-2.08)	0.6		
Absence	53				
CRP (mg/dl)					
>0.4	21	1.39(0.72-2.69)	0.32		
≤0.4	61				
Alb (g/dl)					
>4	41	1.40(0.80-2.46)	0.24		
≤4	41				
CRP/Alb					
>0.089	26	2.24(1.25-4.01)	0.007	3.00(1.58-5.69)	<0.001
≤0.089	56				
ALT (IU/L)					
>30	34	1.28(0.73-2.25)	0.39		
≤30	48				
Variable		Univariate		Multivariate	
	N	HR (95% CI)	p-value	HR (95% CI)	p-value
Liver cirrhosis					
Presence	11	1.35(0.67-2.79)	0.41		
Absence	71				
CEA (ng/mL)					
>5	26	1.27(0.70-2.31)	0.43		
≤5	56				
CA19-9 (U/mL)					
>37	38	1.81(1.03-3.20)	0.039	1.04(0.55-1.99)	0.9
≤37	44				
Lymph node metastasis					
Presence	18	2.98(1.62-5.49)	<0.001	2.64(1.32-5.30)	0.006
Absence	64				
Bile duct invasion					
Presence	49	1.58(0.90-2.78)	0.11		
Absence	43				
Vascular invasion					
Presence	37	2.13(1.21-3.74)	0.009	1.42(0.78-2.60)	0.25
Absence	45				
Tumor number					
Multiple	19	2.93(1.57-5.45)	<0.001	3.34(1.69-6.61)	<0.001
Single	63				
Tumor size (cm)					
>5	25	1.70(0.94-3.05)	0.77		
≤5	57				
Surgical margin					
Positive	12	1.63(0.79-3.37)	0.19		
Negative	70				
Adjuvant chemotherapy					
Yes	41	1.15(0.65-2.02)	0.63		
No	41				

HR, hazard ratio; CI, confidence interval; CRP/Alb, C-reactive protein/albumin; BMI, body mass index; ALT, alanine aminotransferase; CEA, carcinoembryonic antigen; CA19-9, carbohydrate antigen 19-9

**Table 3:** Prognostic Factors for Overall Survival

Variable			Univariate				Multivariate			
	N		HR (95% CI)		p-value		HR(95% CI)		p-value	
Gender										
Male	60		0.71 (0.37-1.33)		0.28					
Female	22									
Age (year)										
>65	48		1.27 (0.70-2.32)		0.43					
≤65	34									
BMI (kg/m^2^)										
>25	25		0.94 (0.50-1.77)		0.85					
≤25	57									
Hepatitis virus infection										
Presence	29		0.97 (0.53-1.80)		0.93					
Absence	53									
CRP (mg/dl)										
>0.4	21		1.52 (0.77-3.03)		0.23					
≤0.4	61									
Alb (g/dl)										
>4	41		0.84 (0.84-2.74)		0.17					
≤4	41									
CRP/Alb										
>0.089	26		2.98(1.57-5.64)		<0.001		3.39(1.72-6.67)		<0.001	
≤0.089	56									
ALT (IU/L)										
>30	34		1.65 (0.92-2.96)		0.095					
≤30	48									
Liver cirrhosis										
Presence	11		1.16 (0.51-2.63)		0.72					
Absence	71									
CEA (ng/mL)										
>5	26		1.20 (0.62-2.30)		0.59					
≤5	56									
CA19-9 (U/mL)										
>37	38		1.83 (1.01-3.31)		0.047		1.20(0.60-2.40)		0.61	
≤37	44									
Lymph node metastasis										
Presence	18		3.36 (1.73-6.56)		<0.001		2.82(1.29-6.19)		0.01	
Absence	64									
Bile duct invasion										
Presence	49		1.42 (0.78-2.57)		0.25					
Absence	43									
Vascular invasion										
Presence	37		2.19 (1.21-3.96)		0.009		1.42(0.75-2.68)		0.28	
Absence	45									
Tumor number										
Multiple	19		2.42 (1.27-4.59)		0.007		2.70(1.34-5.45)		0.005	
Single	63									
Tumor size (cm)										
>5	25		1.25 (0.68-2.31)		0.47					
≤5	57									
Surgical margin										
positive	12		2.29 (1.09-4.81)		0.029		2.13(0.97-4.72)		0.061	
negative	70									
Adjuvant chemotherapy											
Yes		41		0.94 (0.51-1.72)		0.84					
No		41									

## Discussion

The current study demonstrated that compared with patients with CRP/Alb ratio ≤0.089, those with CRP/Alb ratio >0.089 had approximately three-fold higher risk for recurrence and death after hepatic resection for ICC. To the best of our knowledge, this was the first report to analyze the relationship between CRP/Alb ratio and survival outcomes in patients with ICC.

CRP/Alb ratio was considered to be associated with the inflammatory response induced by cancer progression processes, such as tumor growth, invasion, necrosis, hypoxia, or local tissue damage. Previous studies indicated that a high CRP/Alb ratio was associated with large tumor size, tumor depth, microvascular invasion, and lymph node metastasis in gastric cancer, colorectal cancer, and hepatocellular carcinoma (Ishizuka et al., 2016; Shibutani et al., 2016; Saito et al., 2018; Kinoshita et al., 2015). In the current study, CRP/Alb ratio >0.089 was significantly associated with a large tumor size and bile duct tumor invasion and tended to be associated with vascular invasion in patients with ICC. The cause of increased poor prognostic risk in patients with CRP/Alb ratio >0.089 is not clear. However, given the higher frequency of a large tumor size and vascular invasion, cancer progression may contribute to increase the poor prognostic risk in patients with the CRP/Alb>0.089.

In this study, the serum level of CRP was not associated with the increased risk of recurrence and death. Conversely, Lin et al reported that an elevated preoperative CRP level was associated with poor survival outcomes in patients with ICC (Lin et al., 2016). A possible reason of this discrepancy was the different background inflammatory status of the patients. In the study by Lin et al., (2016) the median tumor size was 5.5 cm in diameter, bile duct tumor spread was observed in 63%, and the mean CRP level was 1.52 mg/dL. Conversely, in this study, the median tumor size was 3.5 cm, bile duct invasion was observed in 48%, and the median CRP level was 0.12 mg/dL. Therefore, the less inflammatory status of the liver in this study population than that in the previous report may have diminished the prognostic impact of CRP elevation alone. Several previous studies indicated that the CRP/Alb ratio was a more valuable prognostic marker, compared with other systemic inflammatory markers, such as the modified Glasgow prognostic score (Shibutani et al., 2016; Wei et al., 2015; Kinoshita et al., 2015). Similarly, combining the values of CRP and Alb into a CRP/Alb ratio may be more useful than CRP alone in predicting the survival outcomes of patients with ICC.

Previous studies identified several risk factors for the development of ICC, such as primary sclerosing cholangitis, liver fluke infestation, hepatolithiasis, hepatitis virus, 1,2-dichloropropane and dichlorometane (Bridgewater et al., 2014; Kubo et al., 2014). These risk factors are considered to induce chronic inflammation in the biliary epithelium and potentially lead to cancer development. In this study, viral or alcoholic hepatitis was identified in 42.7% of all patients with ICC. However, there was no significant difference in the proportion of patients with viral hepatitis or ALT >30 between the CRP/Alb ratio >0.089 and ≤0.089 groups. Therefore, CRP/Alb ratio may not be associated with background liver inflammatory status and carcinogenic potential in liver.

The effective systemic chemotherapy for ICC has not been established because of limited patient numbers in previous reports. Although no standard adjuvant chemotherapy had been adopted in patients with ICC, several reports indicated that gemcitabine or S-1 after surgical resection in patients with biliary tract cancer improved OS (Yoshida et al., 2012; Yamanaka et al., 2014). Moreover, a recent randomized controlled study demonstrated favorable OS in patients with advanced biliary tract cancer after receiving cisplatin plus gemcitabine than gemcitabine alone; in that study, subgroup analysis of patients with ICC showed survival benefit with the combination arm (Valle et al., 2010). Therefore, such regimens of chemotherapy may be possible options for adjuvant treatment in patients with CRP/Alb ratio >0.089.

The current study had some limitations. First, it was retrospective, had a small sample size, and was single-center in design. Second, we decided the cutoff value of CRP/Alb ratio at 0.089, based on a cutoff finder on small patient number. However, the median cutoff value of CRP/Alb ratio reported in one meta-analysis for the subject of several cancers was 0.095 (Xu et al., 2017). Therefore, we considered the approximated cutoff value in this study as appropriate.

In conclusion, in patients with ICC, the preoperative CRP/Alb ratio was an independent predictor of postoperative recurrence and death and may help in the selection of appropriate therapeutic strategies. 
